# Sweet Violet (*Viola odorata* L.): Botanical, Horticultural, Pharmacological, and Economic Aspects

**DOI:** 10.3390/plants15182747

**Published:** 2026-09-08

**Authors:** Mihael Kušen, Aleš Vokurka, Vesna Židovec

**Affiliations:** 1 Department of Ornamental Plants, Landscape Architecture and Garden Art, Faculty of Agriculture, University of Zagreb, Svetošimunska cesta 25, HR-10000 Zagreb, Croatia; mkusen@agr.hr (M.K.); vzidovec@agr.hr (V.Ž.); 2 Department for Plant Breeding, Genetics and Biometrics, Faculty of Agriculture, University of Zagreb, Svetošimunska 25, HR-10000 Zagreb, Croatia

**Keywords:** *Viola odorata*, distribution, taxonomy, morphology, anatomy, medicinal use

## Abstract

Historically, sweet violet (*Viola odorata* L.) was an important plant used in various ways, including folk medicine, rituals and festivals, as food, and as an ornamental plant in gardens. The use of this species has varied from ancient times to the present; it is most consistently praised as a medicinal plant, while its popularity as an ornamental and edible species has been intermittent. A review of the extensive scientific literature on its pharmacological and medicinal properties demonstrates its value and the immense diversity of its uses in these fields to this day. While providing a concise overview of its importance for medicinal applications, this paper focuses on the much smaller body of scientific literature concerning horticulture and botany. The aim is to present a comprehensive overview of research on sweet violet, with a primary focus on combining, connecting, and making more accessible notable and related works concerning its taxonomy, morphology, anatomy, and horticultural production. A review of published studies shows fragmented and too specific studies of anatomical structure that are difficult to compare, as well as data gaps in areas of cultivation, hybridisation, selection, and broader comparisons between different populations of this species. It also highlights the overall economic importance which, in combination with the lack of commercial production, results in potentially problematic practices regarding the exploitation of this species in its natural habitats.

## 1. Introduction

The sweet violet (*Viola odorata* L.) has been known in horticulture and folk medicine since ancient times [1]. Although its importance as an ornamental species is considerable, its potential as a medicinal plant is even more widely recognised. This is clearly evident from a search for scientific papers with the phrase “*Viola odorata*” in the title, where, of over 300 such papers in the Google Scholar [2] database, more than 80% are directly focused on pharmacology and medicine. There are very few works regarding this species from other fields such as ecology, morphology, anatomy, physiology, and horticulture, and those are also narrowly focused thematically. Therefore, the aim of this paper is to provide a broad overview, combining and comparing scientific works on similar topics dealing with the species *Viola odorata*, mostly from the fields of botany and horticulture.

### 1.1. Methodology

This study was conducted as a narrative review providing synthesis of the available scientific literature on major aspects of research concerning *Viola odorata* L. The selected literature includes experimental studies and key review papers, as well as a small number of books related to its taxonomy, ecology, morphology, anatomy, horticultural production, phytochemistry, medicinal and horticultural use, both modern and traditional.

The literature was primarily identified and retrieved through Google Scholar, ScienceDirect, PubMed and Web of Science, using phrases such as “*Viola odorata*”, “sweet violet”, “*Violaceae*”, “*Viola*” and “*V. odorata*” in combination with keywords like “taxonomy”, “morphology”, “anatomy”, “medicinal”, “pharmacology”, “cultivation” and “horticulture”.

No restrictions on year of publication were applied; therefore, both recent articles and older studies were included to ensure the most comprehensive coverage of the information. Emphasis was placed on recent research, but earlier foundational studies were included where necessary to provide additional background. Non-English publications were rare but were not excluded if they addressed unique topics and could be reliably translated. Some books written in Croatian were used primarily for morphology and anatomy because they are among the most recent publications, presenting and explaining the latest insights and terminology in those fields. Sources lacking scientific validation, articles without full-text availability and duplicate publications were excluded.

For screening of topics in the fields of pharmacology and medicinal use (because of the large number of specific topics covered), review papers were used as the primary source, together with their most prominent references for further clarification. Screening of papers in the fields of botany and horticulture was detailed and included all the literature identified in the initial search. Studies not directly related to species of the genus *Viola*, and not presenting relevant connections or insights for *Viola odorata*, were considered only for the introduction segment of the paper or used to highlight the lack of research on the species in question.

### 1.2. Scope and Methods of Up-to-Date Botanical Research

During the 20th century, numerous taxonomic studies were published on specific groups within the genus *Viola*, mainly based on morphology [3] and chromosome number [4,5,6]. Research of plant morphology has advanced over time through analytical approaches, new technologies, and tools, but morphological studies are still undervalued and marginalised [7], although comparative morphology can provide significant insight into systematics, reproductive strategies, and ecological adaptation [8]. Nonetheless, comparative morphological studies on violets are almost always combined with other disciplines, primarily anatomy and physiology [9,10], molecular methods [11,12,13,14], and chemical analyses [15,16]. However, unlike morphology, there are studies [17,18,19] that deal solely with the anatomical structure of the sweet violet.

Karyotypic analyses of the genus *Viola* have enabled the reconstruction of the origin of hybrid polyploid species. The study of hybrid populations shows that hybridisation, including backcrossing, is common and that mechanisms exist to balance odd-numbered karyotypes, thereby circumventing sterility [20]. Allopolyploidy has proved to be the main source of the development of new species and the differentiation of species, which took place mainly during the Oligocene cooling period about 30 million years ago [21]. This is stated by Marcussen et al. [22], through their reconstruction of the phylogenetic polyploid network of species within the genus *Viola*, with which they timed and estimated that polyploidy affected 67–88% of cases of the emergence of new species.

There have been various taxonomic classifications of groups within the genus *Viola* [3,6], but more recent molecular phylogenetic analyses [23] have clarified the composition and relationships and re-evaluated the placement of controversial groups. In the 21st century, phylogenetic evidence suggests a division into two subgenera and 16 sections worldwide [22], or 31 sections according to the latest revision [24].

Molecular markers have been little used in research on sweet violet, and data on genetic structure and variability are limited. The basic methods of molecular analysis previously used to assess the variability of sweet violet have been protein isozyme markers [25,26]. Molecular markers based on DNA molecules have been used to assess genetic variability and population genetic structure in other species of the genus *Viola*. The most commonly used are RAPD [9,14,27], ISSR [28,29], and AFLP markers [30,31,32,33]. To determine genetic diversity in crop species, many studies use SRAP markers based on the PCR method [34]. Research on other species has shown the importance of AFLP markers for investigating variability between and within species, mainly due to their multilocus character and assumed genome-wide distribution [31], despite their ambiguity with codominant markers in polyploids [35].

Research into growing conditions aims to verify different methods that can increase productivity, such as plant mass, improve chemical composition, or enhance resistance to certain unfavourable conditions. Various factors under investigation for *Viola odorata* include the influence of salt stress on the accumulation of specific ions, organic compounds, and enzyme activity [9]; the effect of magnetic field strength on growth and chemical composition [36]; the induction of tetraploidy to improve phytochemical and medicinal properties of sweet violet [37], as was found effective for other medicinal species [38,39]; and the impact of mycorrhizal fungi and bacteria on the development of sweet violet [40]. In particular, conditions affecting the success of tissue culture propagation are also being studied, such as the influence of growth regulators [41], the combination of salt stress and drought [42], and various hormones [43] on the quality of the resulting callus tissues and plant organs.

## 2. Taxonomy

Classification [44]: Kingdom: *Plantae*; Phylum: *Tracheophyta*; Class: *Magnoliopsida* (*Dicotyledonae*, *Magnoliatae*); Order: *Malpighiales*; Family: *Violaceae*; Genus: *Viola* L.; Subgenus (subg.): *Viola*; Section (sect.): *Viola*; Subsection (subsect.): *Viola*; Species: *Viola odorata* L.

Synonyms [45]: *Viola gonzaloi* Sennen; *Viola martia* Gilib.; *Viola parmensis* Bailly; *Viola tenerrima* Halácsy & Braun; and *Viola thomsonae* Chapman.

Common names [45]: sweet violet, fragrant violet, common violet (English); Violette odorante (French); *Viola* mammola (Italian); Wohlriechendes Veilchen, Duftveilchen (German); and Banafsha (India).

Within the family *Violaceae* Batsch, the genus *Viola* L. with its sister genus *Noisettia* Kunth forms a strongly supported and distinct clade [46]. Genus *Viola* is the largest, and the number of species has changed most significantly in the last 30 years. In the first revision of the genus in 1925, Wilhelm Becker established that it had about 400 species [3,24]. Beattie and Lyons in the year 1975, as shown in Ref. [47], attributed about 300 species to the genus *Viola*; 20 years later, Erben [20] stated there were about 450, and most published papers in the past 30 years assumed that the genus contained between 525 and 620 species [14,18,31,48,49,50]. In the latest work presenting a revised phylogenetic classification, Marcussen et al. [24] recognised 664 species within the genus *Viola*. According to the WCVP [45], the region most abundant with native species from *Viola* genus is the region of Temperate Asia (262 *Viola* species), followed by Africa (177), South America (159), Europe (159), North America (117), Tropical Asia (51), Australasia (16), the Pacific (7) and Antarctic (3).

The genus is divided into two subgenera: *Viola* and *Neoandium*. The first, to which the sweet violet belongs, is distinguished, among other features, by young (incompletely developed) leaves with curled edges, flower stalks often longer than the developed leaf lamina, and the most common development of cleistogamous flowers [24]. The entire genus is divided into 14 or 16 sections according to various authors [14,22,23,31], a division that began a hundred years ago based on morphology [3]. However, with new insights into species diversity, the genus was most recently divided into 31 sections according to the new phylogenetic revision [24].

Section *Viola*, one of the largest groups of violets in Europe [18], is traditionally divided into five subsections: *Viola*, *Rostratae* Kupffer (including *Repentes* (Kupffer) W. Becker), *Stolonosae* Kupffer, *Adnatae* W. Becker, and *Boreali*-*Americanae* W. Becker [22,51], with the last represented in Europe only by the non-native species *V. sororia* Willd., native to North America.

Subsection *Viola* includes approximately 25 species native to temperate zones of Eurasia and parts of North Africa [25,26]. A most reliable distinction of this subsection is the unique morphology of the fruit: capsules round, non-explosive, on flattened stalks [25,26,31]. However, it can also be distinguished from other subsections within section *Viola* by combinations of the following features: absence of a leaf-bearing stem above ground, presence of a short and thick rhizome, peduncles (which may be reduced or absent), free stipules, nearly rounded lobes, blunt or bluntly acuminate at the apex, beak-like shape of the pistil, and ovoid seeds with a prominent elaiosome adapted to myrmecochory [31,51,52,53,54].

*V. odorata* has been characterised as a species with relatively little morphological variation in its natural range [31]. Most of the infraspecific taxa that have been described are geographically restricted and poorly differentiated, such as var. *maderensis* (Lowe) Webb from the Canary Islands and Madeira, and *V. stolonifera* Rodr. from Menorca [26]; according to GBIF [55], the accepted name is *Viola odorata* subsp. *stolonifera* (J.J.Rodr.) J.J.Orell & Romo). The Caucasus violets seem to differ somewhat more from the European populations in some morphological features, and it is possible that this geographical race deserves taxonomic recognition [26].

The World Checklist of Vascular Plants [45] lists nine accepted hybrid species resulting from naturally occurring crosses between sweet violet and seven different species of the genus *Viola*. Hybrids arising from crosses with species from the *Viola* and *Rostratae* subsections are presented in Table 1.

## 3. Distribution and Ecology

The genus *Viola* is widely distributed in temperate habitats of the Northern Hemisphere, often occurring in higher mountains near the equator and in the Southern Hemisphere. The genus, like the entire family, is thought to have originated in South America [24], but recent centres of morphological and taxonomic diversity are found mainly in the Northern Hemisphere [23,48]. Morphological epicentres of diversity occur in the Alps, the Mediterranean, the Himalayas, and the mountainous regions of East Asia, as well as in the east-central temperate forests of North America, the mountains of Mexico, and the Andes of South America. In contrast, the continents of Africa and Australasia are home to only a few species [50,56,57].

The sweet violet (*Viola odorata* L.) is native to southern Europe, north-western Africa, and western Asia [26]. Due to its use for medicinal and cosmetic purposes, it has spread beyond its area of origin, where it was extensively cultivated [25]. As a result of numerous introductions outside its natural range, it can now be found in temperate climates on almost all continents [26]. According to recorded observations in the “Flora Croatica Database”, sweet violet is widespread in all biogeographic regions of Croatia (lowland continental, mountainous, coastal) [58]. Due to such a wide distribution area, even in the case of Croatia, it can be concluded that this species is highly adapted to different environmental conditions for growth and development, which are determined by the high diversity of soil, climate, and relief of the observed area.

Sweet violet is a perennial, herbaceous, evergreen [40,41,59] and rosette species (*rosulate*), which does not develop lateral aerial stems with leaves and flowers (*acaulescent*), but instead produces runners (*stoloniferous*) that are thin, long, and can easily root upon contact with the soil. Flowers may be chasmogamous (open, cross-fertilised, entomophilous) or cleistogamous (closed, self-fertilised). In the genus *Viola*, chasmogamous flowers are visited and pollinated primarily by solitary bees (*Anthophoridae*, *Halictidae*, *Andrenidae*; *Hymenoptera*), bumblebees (*Apidae*, *Hymenoptera*), hoverflies (*Syrphidae*, *Diptera*), bee flies (*Bombyliidae*, *Diptera*), butterflies (*Lepidoptera*), and, in some species, beetles (*Coleoptera*) [47,60,61]. The alternation of chasmogamous and cleistogamous flowers in sweet violet is regulated by photoperiod, with the development of chasmogamous flowers induced at day lengths of 11 h or less [62]. In many species of this genus, a reserve mechanism of delayed self-fertilisation is also present in chasmogamous flowers, whereby if timely cross-fertilisation does not occur, self-pollination takes place towards the end of flowering [60,63].

For some species of the genus *Viola*, it has been determined that the temporal transition to the development of cleistogamous flowers correlates with a decrease in light due to canopy closure after leafing [60]. Cleistogamous flowers of sweet violet develop under favourable conditions during the summer months, i.e., with a day length of 14 h or more, and their development period is a quarter shorter than that of chasmogamous flowers [62]. The seeds are particularly large in the *Viola* subsection, which is uniquely characterised by obligate myrmecochory [47], facilitated by the presence of an elaiosome that covers up to half the length of the raphe (seed ridge/seam). In the *Viola* section, seeds of chasmogamous flowers have been observed to be larger and heavier than those of cleistogamous flowers, and in sweet violet they are up to twice as heavy [64].

Many sources recognise the difficulty of delimiting species in the genus *Viola*, and Scoppola et al. [8] state that for delimiting sweet violet, a critical taxon of the genus, morphology alone is insufficient. A common feature of violets (*Viola* spp.) is the tendency to hybridise and transfer genetic material due to the lack of reproductive barriers between related species [24]. This is especially pronounced in disturbed and transitional habitat types where such species come into close contact [65,66]. This is often the case with fragrant species of this genus (*V. alba* Besser, *V. suavis* M.Bieb., and *V. odorata*) [11], in which identification is then extremely difficult [25,66]. Hybrids and variable individuals can be further maintained by cleistogamy and vegetative propagation, common adaptations of these species [11]. Considerable variation in traits among populations of certain species of the genus *Viola* (including *V. odorata*) has been demonstrated by Ghorbani et al. [67], who suggest that such diversity could be due to high genetic potential among populations, differences in environmental conditions, or population–environment interactions.

Sweet violet is very prone to hybridisation, and there are even intermediate types of interspecific descendants with transitional morphology between lateral stems and runners (*V. odorata* × *V. riviniana*) [24], which also indicates the homology of these organs. In some regions, alongside sweet violet (2n = 20), one of its most closely related species, *Viola suavis* M.Bieb. (2n = 40), also occurs. Although rare, there are recorded cases of their crosses that are not completely sterile, which is actually common with interspecific crosses within the section *Viola* [26]. In violets of the “Parma group”, characterised by full flowers due to transformed reproductive organs resulting in sterile chasmogamous flowers, the achievement of variability relies only on the occurrence, observation, and further vegetative propagation of shoots in which bud mutation has occurred. However, even in such violets, small fruits with seeds can develop in cleistogamous flowers because petal development is significantly suppressed [68]. The ability of sweet violet pollen to germinate easily on media lacking or oversaturated with glucose compounds, according to Pădureanu [69], demonstrates that the species has very high ecological plasticity. Such ecological plasticity has resulted in this species having high resistance to various weather conditions during flowering across a wide distribution area, from Europe, Asia Minor, Syria, and the Caucasus to North America [69]. Sweet violet exhibits a type of weak physiological seed dormancy, and the hard seed coat also acts as a physical barrier that prevents embryo development [70,71].

*V. odorata* is a common species occurring from lowland to pre-mountainous zones on various soils; however, not all species of this genus display such a wide altitudinal distribution [16,72]. Bayramova et al. [72] recorded stable populations at altitudes between 930 and 1500 m a.s.l. on moist forest soils and in populated areas. In the Himalayas, it is found at altitudes between 1500 and 1800 metres [41]. Regarding soil, sweet violet prefers rich, moist soils with a neutral pH [73], although it can be found on extremely heterogeneous soils with pH values from 4.34 to 7.03 [74]. The common characteristic of those soils is that they are well supplied with humus (3.52–10.86%) [75]. Due to its habitat, sweet violet is often exposed to prolonged daily sunlight, but it appears to have developed sophisticated defence mechanisms, including structural, biochemical, and physiological adaptations to combat UV-B radiation [76].

In areas where it occurs as a naturalised species, it can be found in habitats created and influenced by humans, such as parks and cemeteries, as well as in natural and semi-natural open dry grasslands, shrubs in shaded alluvial forests [31,72], in hedgerows, and along forest edges [77]. This is confirmed by Adamowski [78], who adds that the species is still often found in tended garden lawns, as well as in neglected gardens and plots, along fences and roads, but it does not occur in old and well-preserved forests.

In certain parts of the world, especially Western Asia (Iran), sweet violet is an important medicinal plant and is overexploited [42]. Due to its use in folk medicine for various herbal preparations, and in the absence of commercial cultivation, its abundance in that part of its natural distribution area is rapidly decreasing, and without conservation measures it could become endangered [41,43].

## 4. Morphology

### 4.1. Vegetative Parts

Sweet violet is a rosette-shaped, herbaceous species, reaching up to about 15 cm (7–22 cm) in height, with a creeping, broadly cushion-like habit due to numerous thin shoots [72,73]. The roots are thick at the upper part [79]; in natural habitats, the length and branching of the roots vary depending on soil type. In lowland areas with richer soils, the root is shorter and highly branched, while in pre-mountainous areas on skeletal and poor soils, the root is long and less branched [16]. The rhizome is formed from the stem of main and lateral rosettes; it is short, up to 2 cm in older plants, thick, initially whitish, and becomes darker with age [72].

Sweet violet is one of the species within the subsection *Viola* that can be distinguished by the presence of runners, which is a much more reliable characteristic than in other species. Research by Hodalova et al. [31] showed that, unlike *V. alba* ssp. *alba* and *V. suavis*, in which up to a third of the plants may lack runners, only 8% of sweet violet plants are found without runners. The runner develops from young rosettes and/or from rhizomes on older plants; it is long and thin [80] and roots upon contact with the soil [11].

During development, young leaves are folded (with curled edges) towards the adaxial side of the lamina, along the mediolateral plane [63]. Fully developed leaves are round or sometimes almost kidney-shaped, deeply toothed near the base of the lamina, dark green, matte, and borne on long petioles, which are densely hairy in the upper half [11,72,79].

The petioles of this species are oval to lanceolate, smooth-edged or with short glandular appendages (*fimbriae*) along the margin [11,31]. The flower stalk has bracteoles that can serve as a distinguishing characteristic between some species of the genus; in sweet violet, their position is most often on or above half the stalk [11,52,72,81], but they can also be observed below half the stalk [31]. A summary of morphological traits is given in Table 2.

### 4.2. Generative Parts

The flower stalk is leafless and sharply curved at the top, resulting in a flower directed towards the ground [41,77]. In sweet violet, the sepals are characteristically rounded to bluntly pointed [80] and bear short basal appendages (so-called spurs) [63].

**Figure 1:**
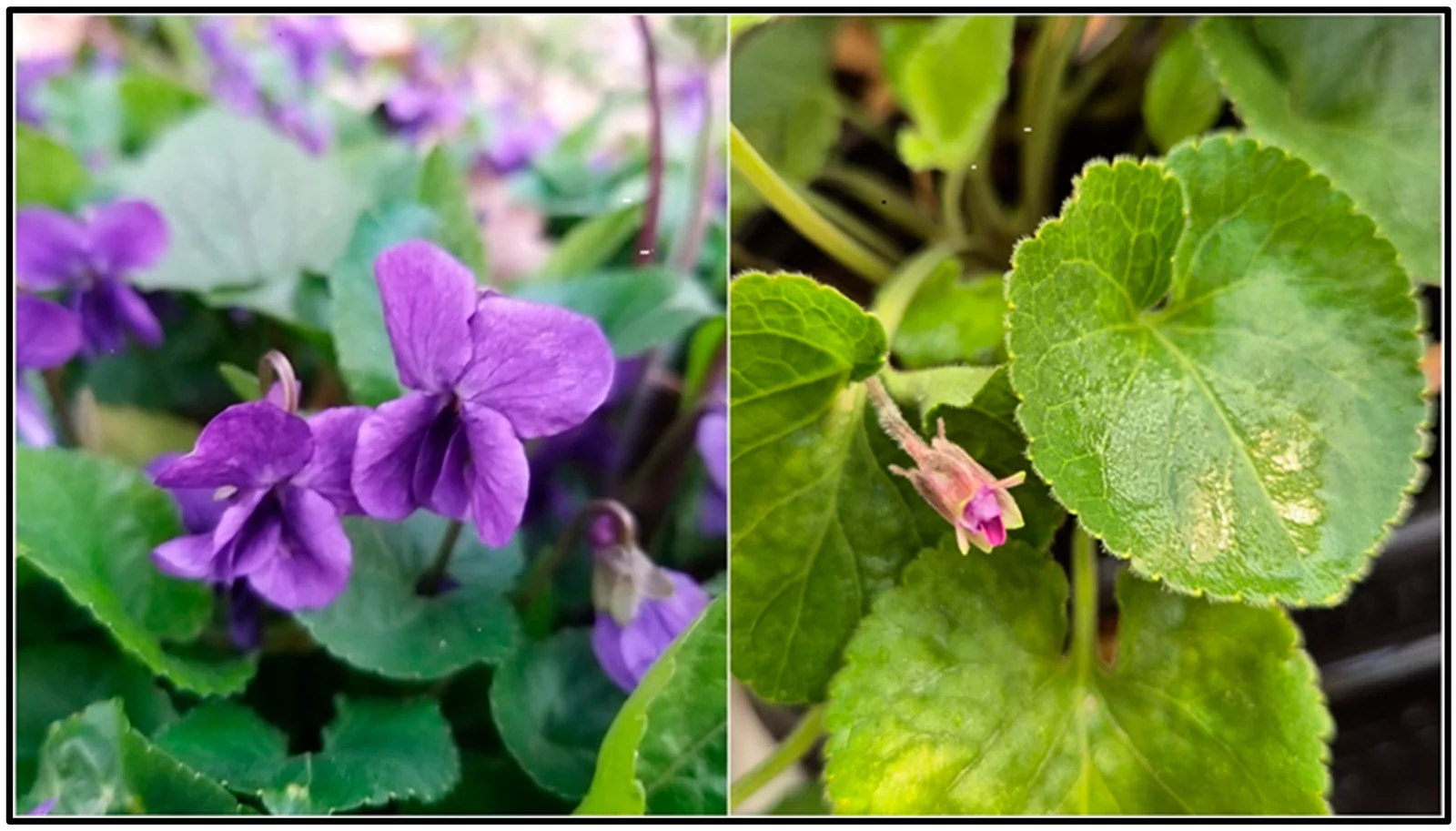
Chasmogamous spring flowers (**left**); cleistogamous summer flower (**right**).

The flowers are solitary, lateral, form a floral rosette, and display a typical light or dark purple colour, although they are sometimes white [11,26]. They can also be intensely dark purple, sometimes with a bluish-white base to the petals [72,79]. The chasmogamous flower (Figure 1) has a uniaxial symmetry (zygomorphic), which developed with the formation of a petal spur located on the petal forming the lower lip. The spur does not contain nectaries, but stores nectar produced by the basal nectary appendages of the stamens [63].

At maturity, the petals of cleistogamous flowers are often colourless and membranous, remaining enclosed within the calyx (Figure 1) [82]. Interestingly, in cleistogamous flowers, there is a dorsal facial reduction of the anthers so that only those closer to the centre of the flower remain on the filament [63]. The fruits are non-explosive capsules with a purple tint or purple spots, and develop on horizontally lying stalks [77]. The seeds are obovate and smooth, 1.5–1.8 mm long, with a width and thickness of up to 1 mm [72].

## 5. Anatomy

### 5.1. Vegetative Parts

A cross-section of the root shows structures characteristic of secondary growth. The outer part of the cortex (exoderm, rhizoderm) contains three to four rows of well-formed cells. The cells are thin-walled, broad, and mostly smooth. Calcium oxalate crystals are randomly distributed among the parenchymal cells of the cortex [17,49]. The vascular tissue in the secondary structure consists of approximately eight to nine rows of flattened secondary phloem on the outside and circular secondary xylem on the inside. The secondary xylem has small cells, and the primary xylem is a wide trachea. The central part of the vascular cylinder contains non-lignified parenchyma with relatively large, thick-walled cells in which the primary elements are embedded [17,49].

In sweet violet, the cross-section of the main stem is irregularly square [83], while the cross-sections of the lateral branches (runners) are circular, lacking the wing usually present in other species of the *Viola* subsection [18]. The epidermis consists of one to two layers of collenchyma cells, and simple, unicellular stipules are located on the surface [18]. Below the collenchyma layer is the parenchyma zone with round cells and intercellular spaces. The central area of the lateral branches consists of spherical parenchyma cells that store calcium oxalate crystals [17,49].

The general shape of the petiole is heart-shaped due to the two lateral wings. The epidermis, consisting of a single row of round cells, is surrounded by a thick cuticle [17]. The angular collenchyma is located immediately below the epidermis and consists of two to three rows of cells. After the collenchyma, there is a parenchyma layer consisting of four to nine rows of round, thin-walled cells; the central area is not empty, and calcium oxalate crystals can be observed within it [17,18]. The vascular bundle is arranged between single-rowed and double-rowed sclerenchyma. There is an open collateral type of vascular bundle. The cambium between the xylem and phloem is thin and has one to two rows of cells. The epidermal cells of the petiole bear covering hairs [49].

The lower and upper epidermis are covered with a cuticle layer. Cylindrical or conical hairs are unicellular and develop in the midrib zone on the adaxial side of the leaf [18], while multicellular glandular hairs are located on the teeth along the edge of the lamina [19]. Parenchyma cells are found on the surfaces of the vascular bundles directed towards the upper and lower epidermis. Stomata are present in both the upper and lower epidermis (amphistomatic leaf). Epidermal cells do not differ in size on the upper and lower sides of the leaf [49]. In the cross-section, in the middle of the leaf, a midvein area is visible, in the centre of which open collateral vascular bundles are located, surrounded by parenchyma and oval cells on all sides. The stomata are of the anisocytic type; the stomatal index of the upper surface is 4.0, while that of the lower surface is 30.7 [19]. Changes in the anatomical structure of the leaf may occur due to increased UV-B radiation at exposed positions, increasing the number of mucous cells and mucous channels, the thickness of the walls of epidermal and subepidermal cells, and the diameter of the vascular bundles [76].

### 5.2. Generative Parts

The cross-section of the flower stalk is square, with the outer layer consisting of a single layer of oval cells covered by a cuticle. Between the parenchyma centre and the epidermis is a single layer of sclerenchyma, which becomes double-layered towards the corners of the stalk [17]. In the sweet violet, there are also cells in the stalk that store crystals. In the central part, there are four collateral conductive bundles separated by up to two layers of round parenchyma cells [17].

The scent of chasmogamous flowers is produced by idioblasts in the epidermis and mesophyll of the petals, which contain volatile essential oils that evaporate through the cell walls and cuticle [63]. The flowers are described as intensely fragrant [11,26] or sweetly fragrant [72,79] due to their volatile organic compounds, and the amount and composition of intensely fragrant compounds vary among cultivars of sweet violet [84]. The nectaries of this species are present as connective appendages between the two lower stamens and are composed of three layers: epidermis, nectary parenchyma, and subnectary parenchyma [73]. The pollen, in equatorial view, is prolate-spheroid to subprolate; the ornamentation of the exine shows that the sweet violet has a perforated to subpsillate and perforated to granular exine [18].

## 6. Chemistry and Pharmacology

A study of the chemical composition of the stem, leaves, petiole and flower of the sweet violet showed that this species is a rich source of elements such as carbon, oxygen, sodium, calcium, magnesium, aluminium, silicon, chlorine and iron [85], and that phosphorus and potassium accumulate in different parts during the season [59]. Several studies have documented compounds isolated and identified in essential oils obtained from different species of the genus *Viola*, with the largest number of isolated compounds, as many as 47, found in the sweet violet [86]. The greatest diversity of phenolic acids and flavonoids was also found in the sweet violet [83]. The nutritional profile revealed that different parts of the plant contain fats and oils, proteins and starch [87], and various groups of phytochemical compounds such as saponins, glycosides, terpenoids, flavonoids, steroids and alkaloids have been isolated from the sweet violet [88,89].

All parts of the plant contain a wide range of compounds, including alkaloids, glycosides, saponins, methyl salicylate, mucilage, vitamins including vitamin C, *Viola*ntin, flavonoids, stigmasterol, *Viola*quercetin, peptides, phenols, glucosides, *Viola*cin, violin, *Viola*ntin and *Viola*nin, vanillic acid, benzofuranone, shikimic acid, cyclo*Viola*cin, dimethyldodecane, dimethylheptane and glucopyranoside [79,90,91]. Salicylic acid is found in all parts except the rhizome; stigmasterol is found exclusively in the leaves [87], and the flowers also contain anthocyanins and rutosides [92].

Numerous cyclotides discovered in plant families are predominantly found in the family *Violaceae*, with many newly discovered in the species *V. odorata* [93]. Cyclotides in plants act as a natural defence mechanism and exhibit various biological activities that could be applied in agriculture and pharmaceuticals [94]. The compounds cyclo*Viola*cin O2, O3, O8, O13, 14, 15 and 16, isolated from the sweet violet, have also been identified as insecticidal agents [86,94]. The essential oil of this species has a repellent effect against Culex, Aedes and Anopheles mosquito strains [95], and the essential oil of the flowers also has fungicidal properties [96]. High percentages of monoterpenes and sesquiterpenes, phenyl butanone, linalool, benzyl alcohol, α-cadinol, globulol and viridiflorol are obtained from the essential oil of sweet violet flowers [96]. The essential oil of sweet violet leaves contains butyl-2-ethylhexylphthalate and 5,6,7,7a-tetrahydro-4,4,7a-trimethyl-2(4H)-benzofuranone [97]. Orhan et al. [98] suggest that the inhibitory effect of the alcoholic extract on tyrosinase can be attributed to its flavonoid derivatives, vitexin, isovitexin and kaempferol, which could be applied in cosmetics for the treatment of skin discolouration. Essential oils from flowers and leaves are also used in aromatherapy [99], while aqueous or alcoholic extracts can be used for the biosynthesis of metal oxide (NiO) nanoparticles which have versatile biological and therapeutic properties making them suitable for biomedicines [89].

## 7. Propagation and Cultivation

Sweet violet is known as an ornamental species [1,37,40,68], but today it is rarely found as a species intentionally planted in gardens [78]. Despite its demand, primarily due to its medicinal value, production remains significantly low, partly because of low germination resulting from seed dormancy [40,82], mainly due to the hard seed coat [71]. The summary of propagation and cultivation methods are given in Table 3.

Dormancy breaking is effectively achieved by cold stratification at 4 °C for 8–12 weeks, especially with gibberellic acid treatment [100]. Concentrated sulphuric acid was also found effective, and dormancy can be overcome by endosperm and embryo culture [101]. Seed scarification by removing the top of the seed coat has proven to be an effective and simpler method of promoting germination, especially in combination with cold stratification and gibberellic acid, which also promotes faster swelling and better subsequent development of the plant after germination [71]. In commercial production, after sowing in autumn and overwintering in a cold protected or open area, the plants germinate in March but do not flower in the first year [102]. Inoculation with mycorrhizal organisms [40], application of nitrogen fertiliser through an aqueous solution, and foliar treatment with gibberellic acid [10] have positive effects on growth and development.

The most effective methods of vegetative propagation are rhizome division [103], and propagation by stem cuttings [68], which are carried out in July; plants propagated in this way are suitable for planting outdoors or in containers as early as September [102]. Vegetative propagation by stem cuttings was preferred throughout a long period of historical production as the only method that could be used to preserve and obtain new cultivars arising from spontaneous mutations in the apical vegetative buds [68].

The development of tissue culture methods has enabled the efficient production of plants identical to the desired type and the improvement of species using genetic engineering techniques. The importance of tissue culture is increasing, and it has now become a well-established technique for the preservation of rare, endangered, or medicinal plants [104] such as the sweet violet. Research by Haralkar and Biradar [103] showed that using explants of leaves and petioles in a growing medium enriched with several combinations of growth regulators can produce large amounts of callus with satisfactory rhizogenesis.

There is limited data on the general conditions of cultivation in commercial production; most scientific papers focus on the absorption and distribution of macro elements in the plant. Insight into the traditional production method in the Toulouse region is provided by Fan and Morard [105], who describe the cultivation of sweet violets as follows: cultivation takes place on sandy alluvial soils containing 4.3% organic matter and a pH (H_2_O) of 7.6; during soil preparation, 500 kg of manure and about 7.6 kg of nitrogen are added per 100 m^2^; in July, additional fertilisation is carried out with a balanced NPK fertiliser (17-17-17) at a rate of 10 kg per 100 m^2^; during cultivation, the runners are removed to avoid reducing flowering, and irrigation is provided. Other, more detailed studies on fertilisation have also highlighted the importance of regular application of complex fertilisers [74,75,106], particularly the significance of phosphorus and potassium during vegetative growth, as these elements are then accumulated and stored in leaves and stems [59]. Unlike many ornamental plant species suitable for cultivation in commercial peat substrates, better growth and development of sweet violets has been recorded when grown in soil collected from the original habitat of the cultivated plants [74], that is, in garden and forest soil which is poorer in organic matter and has a slightly higher pH (7.1–7.7) [75]. Ex situ cultivation should be studied in greater detail, particularly regarding changes in chemical composition that may occur due to the likely elimination of stress factors in commercial production that are present in the wild [107].

**Table 3:** Summary of propagation, seed treatments and cultivation improvement methods.

	Propagation	Reference	Seed Dormancy Braking	Reference	Improving Growth and Development	Reference
Method	Rhizome division	[103]	Cold stratification at 4 °C 8–12 weeks	[100]	Aqueous solution of nitrogen fertiliser	[10]
			Gibberellic acid		Foliar gibberellic acid	
	Stem cuttings in July	[67]	Cold open overwintering	[102]	Balanced NPK fertiliser	[105]
	Tissue culture	[104]	Concentrated Sulphuric acid	[101]	Induction of tetraploidy	[37]
	Explants of leaves and petioles + growth regulators	[103]	Endosperm or embryo culture		Mycorrhizal fungi and bacteria	[40]
	Seed sowing in autumn	[102]	Scarification	[71]	Growth hormones and regulators	[41,43]

## 8. Economic Value

### 8.1. Historical Use

Fragrant violets (*Viola* spp.) have a long history of use in Europe, with the earliest records dating back to Ancient Greece. They were sold in the Athenian agora, praised by Greek poets, used in medicine and for making garlands, and featured in stories and mythology [1]. Since then, the leaves and especially the flowers of sweet violets have been used in medicine and cosmetics, and the species has been cultivated as an ornamental plant in gardens [26]. The sweet violet was and still is best known among all *Violet* species for its medicinal properties. In traditional medicine, all parts of the plant are collected for medicinal purposes, while the perfume and cosmetics industries mainly use the flowers. As early as the 17th century, there are references to making juice, water, syrup, honey, oil, and vinegar from violets, and the candied petals and leaves are used by the confectionery industry [108].

After the highest recognition in the Middle Ages, the violet fell into obscurity until the 19th century, when Kneipp became a prominent advocate [109]. The golden age of cultivating fragrant violets (*Viola* spp.) began in France at the end of the 18th century and in Great Britain at the beginning of the 19th century, when gardeners enthusiastically created new cultivars. The introduction of the robust Russian violet (*Viola suavis*) also supported breeding and industry [108].

The first known cultivars of violets are mentioned in the 19th century, notably the famous Parma violets derived from the species *Viola alba* Besser subsp. *dehnhardtii* (Ten.) W. Becker [68]. By the end of the 19th century, after a disease seriously affected the Parma violets, farmers on the Côte d’Azur began to cultivate the sweet violet, *Viola odorata* ‘Victoria’ [1]. It was grown mainly for the production of essential oils for the perfume industry [110,111]. The sweet violet was among the most popular cut flower species in the first half of the 20th century. For this purpose, a large number of different cultivars were developed in numerous shades, from dark purple, purple, and pink to white flowers, differing also in fragrance intensity, flower size, and petal shape [15]. Its rise and decline in popularity is illustrated by the fact that at the turn of the 19th and 20th centuries, in the French province of Toulouse, over 600 family farms earned at least part of their income from the production of sweet violets, then on a total of 25 ha, but by 1985 that number had dropped to only 7 farms with production on around 0.5 ha [105].

### 8.2. Modern Gastronomy

Edible flowers have emerged as a fascinating and versatile ingredient in culinary and luxury tourism contexts [112]. In addition to their decorative uses, sweet violet flowers have practical applications, such as flavouring liquids and spreads, preserving in sugar or salt, and freezing in ice cubes. The flowers of this species offer many culinary possibilities, making them an appealing addition to special occasions and events where both taste and visual appeal are desired [113]. Among edible flowers, sweet violet is one of the most commonly consumed, with a velvety texture and a sweet, refreshing taste [114]. Nekouyar et al. [10] demonstrated that sweet violet has a higher antioxidant capacity than wild pansy (*Viola tricolor*), which is more commonly produced commercially as an edible flower species. The same authors state that both species are sources of protein, minerals, and antioxidant compounds, and have shown that the edible value can be increased by applying a certain concentration of GA and N, which for sweet violet increases the content of protein (68%), carotenoids (175%), Fe (144%), and Zn (150%) in the petals.

### 8.3. Medicinal Use

*Viola odorata* is an important herb in folk medicine for various diseases such as diabetes, cancer, postoperative tumours, bronchitis, cough [115,116], and common digestive disorders [86]. In traditional medicine, it is used for insomnia, low blood pressure and anxiety [117]. A clinical study conducted by Mehraban et al. [118] showed that the addition of sweet violet syrup has an effect on controlling accompanying symptoms such as cough, fever, myalgia, diarrhoea and headache during COVID-19 infection.

Fresh leaves are used in cancer prevention due to their stigmasterol content [99]. A decoction of dried flowers is used as an analgesic and expectorant, while seeds are used to improve the removal of waste products from the body. An herbal poultice is applied as an analgesic and to treat weakness [119]. The leaves are also used to induce sweating, treat childhood disorders and lung problems, and both leaves and flowers are used for respiratory disorders [120,121]. The stem, flowers, and leaves of sweet violet are used in the treatment of inflammatory, tumour, urinary, liver, and kidney diseases [86,122,123].

*V. odorata* is mainly used to treat skin infections (urticaria, eczema, allergic dermatitis) and as a syrup for coughs and asthma [124]. It is also used for rheumatism and urinary tract infections [121,123], as a pre-anaesthetic, sedative, laxative, antipyretic, and to help with dyslipidaemia and hypertension [90]. Additionally, it can be used to treat catarrh, colds, and insomnia [124]. Among eight bacterial strains tested, cyclo*Viola*cin O2 (CyO2), a cyclotide isolated from *V. odorata*, was found to be the most effective antibacterial cyclotide against *S. enterica* and *E. coli* [125]. Cyclo*Viola*cin O2 also exhibits antitumour effects and causes cell death (cytotoxicity) by membrane permeabilisation [126,127]. The mucilage properties and high content in violet flowers and leaves serve as an emollient and softener, and are therefore used in the preparation of medicines for respiratory and gastrointestinal diseases [128]. All plant extracts show antioxidant activity [129], and aqueous extracts from flowers have demonstrated antioxidant potential in removing potentially cytotoxic radicals [130]. Phenolic compounds that are present in sweet violet usually have strong external antibacterial and antifungal qualities [131], and thus pose potential for use in medicine and agriculture. A summary of recorded medicinal uses from commonly used parts and important compounds is given in Table 4.

### 8.4. Application in Horticulture

Sweet violet is used as a cut flower, which currently has only local and seasonal importance, and only a small number of farms produce it for this purpose [105]. Greater emphasis in horticulture is placed on its use as an herbaceous perennial, its extremely early flowering, and its use in private gardens [78,79,108]. One possible application of this species in horticulture is the stabilisation of slopes [108]. It is ideal for use on gentle slopes in bright, semi-shaded positions and on poorer soils, which it can densely cover, thus requiring little care and maintenance. The species is adaptable and will grow in sunny positions as long as there is sufficient soil moisture [102] during intensive spring growth.

The species can also be grown as a potted plant, and the dense, compact clump of heart-shaped leaves remains decorative all year round [108]. During flowering, the plant is extremely attractive due to the shape, intensity, and variety of flower colours (purple, purple–blue, pink, white, yellow) [78], and especially because of its specific and intense fragrance. Fragrance has been recognised as one of the main components influencing preference for a particular flower species [132]. This long-recognised and valued scent has inspired human activity throughout the centuries and across different cultures [84], and could form the basis for new selection, reintroduction, and popularisation of sweet violet.

## 9. Discussion and Future Directions

Significant new insight into the systematics, reproductive strategies, and ecological adaptations of species in the genus *Viola* has been gained through research over the past 30 years [8,9,10,11,12,13,14,15,16,17,18,19,20]. Progress in technology and methods is evident in the rapid increase in the number of species recognised in the genus *Viola*, resulting from karyotypic analyses and research into polyploidy, bringing us to the current total of 664 *Viola* species divided into 31 sections according to the most recent phylogenetic revision [24]. This number is likely to grow in future years, as DNA-based molecular markers have so far been used to assess genetic variability and population genetic structure in only a handful of species in the genus *Viola* [12,14,27,28,29,30]. With new research clarifying the genetic and epigenetic basis, as well as the confirmed photoperiodic regulation of chasmogamy and cleistogamy in *Viola odorata*, it may become possible to manage the ratio of cross-fertilisation to self-fertilisation for the purpose of fixing lines, which is a classic breeding goal.

*V. odorata* is a critical taxon of the genus, but for proper identification morphology alone is rarely sufficient [8], as even more reliable characteristics such as long, thin stolons (runners) can be absent [31], or appear short under unfavourable conditions. Characteristics such as flower stalk bracteoles should not be considered in the determination process at all, as researchers cannot agree on their position, stating they are located at or above half the stalk [11,50,70,79], but also possibly below half the stalk [31]. The problem of delimiting sweet violet is intensified because it usually inhabits the same areas as similar fragrant species of this genus, with which it frequently hybridises [11], particularly in disturbed and transitional habitat types [63,64]. Infraspecific taxa are for now limited to two geographically restricted records, but there is suspicion that sweet violets from the Caucasus also deserve taxonomic recognition [26]. Therefore, claims that this species shows little morphological variation in its natural range [31] may be disproved by further research, as this species is now so widespread outside its area of origin, occupying habitats with diverse ecological conditions. Further research into the frequently recorded hybridisation of this species could provide new insights into evolution and trait inheritance within the genus *Viola*, and may also yield interesting new hybrid species for selection and horticultural use. New comparative morphological methods, together with additional research into current consumer preferences for ornamental plants, should guide the selection of new cultivars and help restore the former prestige and use of this species in horticulture.

This species demonstrates very high ecological plasticity and strong resistance to various weather conditions during flowering across a wide distribution area [67]. It has also developed sophisticated defence mechanisms, including structural, biochemical, and physiological adaptations to combat UV-B radiation [74]. *V. odorata* has a wide altitudinal distribution between 930 m [16,70] and 1800 m a.s.l. [39], occurring on various soil types with differing pH values from acidic to neutral [72]. It also occurs as a naturalised species, found in habitats created and heavily influenced by humans [31,70,76]. Despite all this, and in spite of its high demand, primarily due to its medicinal value, production remains significantly low [38,80], which suggests that large quantities are being collected directly from the wild. The wide distribution of sweet violet indicates the effectiveness of its breeding strategies and its adaptations to diverse ecological conditions, which could favour the development of more efficient commercial breeding and cultivation methods through further research.

It is often stated as an obstacle in production that sweet violet exhibits seed dormancy [68,69], which does complicate the germination process, but today this should not be used as an excuse for potentially devastating natural populations and endangering this species in specific areas [39,41]. There is now sufficient research and a range of new methods that have proved effective in increasing the success rate of propagating sweet violet by both vegetative and generative means. Dormancy breaking is effectively achieved by cold stratification and gibberellic acid treatment [98], as well as by endosperm and embryo culture and concentrated sulphuric acid [99]. Seed scarification has also proved to be an effective and simple method, especially in combination with cold stratification and gibberellic acid [69]. Vegetative propagation has been shown to be historically reliable, and is the only production method that can be used to preserve and obtain new cultivars arising from spontaneous mutations [66]. The importance of tissue culture is also increasing, as it has enabled the efficient production and improvement of species using genetic engineering techniques, becoming a well-established method for the preservation of rare, endangered, or medicinal plants [102] such as the sweet violet. Commercial propagation and cultivation now only need to be encouraged in order to reduce pressure on wild populations, where there is a risk of overexploitation.

A bigger issue arises from limited data on the general conditions for cultivation in commercial production. Scientific papers that investigate the growing conditions of sweet violet mainly focus on the translocation of macroelements in the plant [57] and the application of complex fertilisers [72,73,104]. There are some very specific studies covering methods that affect growth and development, increase productivity and plant mass, improve phytochemical and medicinal properties, and investigate conditions influencing the success of tissue culture propagation [9,36,37,38,39,40,41]. In addition to existing research on the nutrition of this plant species, further studies are needed on the standard environmental and other cultivation conditions required for profitable commercial production of sweet violet, such as suitable substrate types, temperature, humidity, lighting, and susceptibility to diseases and pests in various modern greenhouse production systems.

Most of the research on phytochemical constituents has been conducted on plant material collected in situ from specific local populations, so there may be variability that is still to be determined across the extremely diverse ecological conditions in which this species grows. Systematic mapping of chemotypes across geographic populations could establish “terroir” characterisation and support the potential protection of indigenous varieties and populations. The scent of chasmogamous flowers, described as intensely [11,26] or sweetly fragrant [70,77], comes from volatile essential oils that evaporate through the cell walls and cuticle [61]. The amount and composition of intensely fragrant compounds from flowers have been shown to vary among cultivars of sweet violet [82], but although present in them, the variability of fragrant compounds from leaves [95,96,97] still remains undetermined. Thus, the scent, one of the most distinctive features contributing to the attractiveness of this species, presents significant potential for further research and forms the basis for previously unexplored selection according to modern principles for this trait.

Changes in chemical composition that may occur due to the likely elimination of stress factors in commercial ex situ production, compared with collection from natural habitats, should be studied in greater detail, as this is an important medicinal plant. However, this is the step that follows characterisation and selection of the most valuable sweet violet populations in the wild and the establishment of cultivation guidelines for sustainable, large-scale commercial production.

## 10. Conclusions

A review of published studies on the sweet violet revealed significant data gaps in fundamental areas such as cultivation, hybridisation, selection, and broader comparisons of the ornamental and medicinal characteristics of different populations of this species. Studies of anatomical structure are highly fragmented in terms of subject matter and focus only on specific plant parts, making them difficult to compare. Morphological studies (now often considered of marginal importance in scientific literature) and research into the scent properties of this species should be conducted on various populations across its native and introduced ranges to gain insight into the species’ potential for new selection and/or hybridisation. There are surprisingly few published scientific papers analysing the general environmental conditions required for applying modern cultivation and commercial production principles—conditions that should provide the foundation for the sustainable use of this species in horticulture, gastronomy, the pharmaceutical industry, and medicine.

## Figures and Tables

**Table 1:** Naturally occurring *Viola odorata* hybrid species.

Hybrid name	*Viola* × *hungarica*	*Viola* × *porphyrea*	*Viola* × *scabra*	*Viola* × *pluricaulis*	*Viola* × *poelliana*	*Viola* × *oeipontana*	*Viola* × *vindobonensis*	*Viola* × *olimpia*
Other parent species	*Viola ambigua*	*Viola collina*	*Viola hirta*	*Viola alba*	*V. collina* × *V. hirta*	*V. hirta* × *V. pyrenaica*	*Viola suavis*	*Viola reichenbachiana*
Subsection	*Viola*	*Viola*	*Viola*	*Viola*	*Viola*	*Viola*	*Viola*	*Rostratae*

**Table 2:** Summary of *Viola odorata* morphological traits.

Vegetative Parts	Habit	Stolons/Runners	Stem/ Rhizome	Young Leaves	Mature Leaves	Leaf Margin	Petioles	Stipules
Description	Rosette, broad cushion	Thin and long	Short and thick	Folded, curled upwards	Rounded, kidney-shaped	Deeply toothed at base	Long, oval, upper half densely hairy	Ovate-lanceolate, fimbriate margin
Generative Parts	Flower Stalk	Flowers	Tepals Shape	Tepals colour	Spur	Sepals	Fruit	Seeds
Description	Leafless, rounded, with bracteoles, sharply curved at the top	Solitary, growing laterally from rosette, commonly above leaves	Rounded, lower middle (lip) ends in spur	Light to dark purple, reddish-purple or white, lighter at base	No nectaries, curved, light to dark purple	Rounded/bluntly pointed, short basal appendages (1–3 mm)	Non-explosive capsules purple tint or spots, on horizontally lying stalks	With elaiosome, obovate and smooth, up to 1.8 mm long and 1 mm thick

**Table 4:** Pharmacology and medicinal use of specific plant parts and important products.

Plant Parts/Specific Product	Recorded Use and/or Constituents Found	References
Fresh leaves	cancer prevention, lung problems	[97]
	stigmasterol	[85]
Flowers	analgesic and expectorant	[117]
	anthocyanins and rutosides	[90]
Flowers and leaves	for respiratory and gastrointestinal diseases	[118,119,126]
	mucilage	[126]
Above-ground parts	inflammatory, tumour, urinary, liver, and kidney diseases	[84,120,121]
	Salicylic acid	[85]
Seeds	removal of waste products	[117]
Herbal poultice	analgesic and to treat weakness	[117]
CycloViolacin O2	antibacterial, antitumour effects, insecticidal	[84,92,123,124,125]
Phenolic compounds	antibacterial, antifungal	[129]
Extracts from flowers	removing potentially cytotoxic radicals	[127,128]
Sweet violet syrup	controlling accompanying symptoms of COVID-19, antibacterial and antifungal qualities	[116]
Leaves and flower essential oils	insect repellent effect, aromatherapy, antioxidant, antifungal	[93,94,95,97]
	monoterpenes and sesquiterpenes, phenyl butanone, linalool, benzyl alcohol, α-cadinol, globulol and viridiflorol, 5,6,7,7a-tetrahydro-4,4,7a-trimethyl-2(4H)-benzofuranone	[94,95]

## Data Availability

No new data were created or analysed in this study. Data sharing is not applicable to this article.
